# Synthesis and structural characteristics analysis of melanin pigments induced by blue light in *Morchella sextelata*

**DOI:** 10.3389/fmicb.2023.1276457

**Published:** 2023-09-29

**Authors:** Zhiheng Qiu, Shuang Wang, Jiazhi Zhao, Lingxiu Cui, Xinyi Wang, Nuo Cai, Hongpeng Li, Shuhua Ren, Tianlai Li, Lili Shu

**Affiliations:** ^1^Modern Protected Horticulture Engineering & Technology Center, College of Horticulture, Shenyang Agricultural University, Shenyang, China; ^2^Key Laboratory of Protected Horticulture of Education Ministry and Liaoning Province, Shenyang, China

**Keywords:** *Morchella sextelata*, blue light, melanin, synthesis, ultrastructural characteristics, color formation

## Abstract

*Morchella sextelata*, a highly sought-after edible mushroom worldwide, is evaluated based on its cap color as an essential commercial property indicator. In the present study, the effects of blue light on cap pigmentation in *M. sextelata*, as well as the synthesis and structural characteristics of melanin pigments within the cap were examined. The results showed that an increase in the proportion of blue light within the lighting environment promoted melanin synthesis and melanization of the cap. Transmission and scanning electron microscopy revealed the localization of melanin within the mycelium and its ultrastructural characteristics. The UV–visible analysis demonstrated that melanin exhibited a maximum absorption peak at 220 nm and possessed high alkaline solubility as well as acid precipitability. The structural characteristics of melanin were analyzed using FTIR, NMR, HPLC, and elemental analysis, which confirmed the presence of eumelanin, pheomelanin, and allomelanin in both brown and black caps. Furthermore, blue light can stimulate the synthesis of both eumelanin and pheomelanin. The obtained results can serve as the foundation for comprehending the mechanism by which light regulates color formation in mushrooms.

## 1. Introduction

*Morchella sextelata*, commonly known as the black morel, is a highly prized edible fungi that is renowned for its distinctive flavor and unique appearance (Kuang et al., 2022). It is one of the many species of the *Morchella* genus, which is a group of ascomycete fungi that are highly sought after by both commercial and recreational mushroom hunters worldwide (Li et al., 2022). *M. sextelata* is a medium to large-sized mushroom that can grow up to 15 cm tall. Its fruiting body is rich in protein, fiber, essential vitamins and minerals, low in calories and fat (Tietel and Masaphy, 2017). Additionally, it has been reported to possess antioxidant, anti-inflammatory, antimicrobial, and immunomodulatory properties attributed to the presence of various bioactive compounds (Meng et al., 2019). Further research on *M. sextelata* has revealed its potential as a source of new bioactive compounds (Chen et al., 2023). The unique morphology and ecology of *Morchella* species have led to the production of a wide range of secondary metabolites, many of which have not been fully characterized.

The cultivation of *Morchella* species is a challenging process that requires a deep understanding of their biology and ecology (Ower, 1982). However, the success of artificial cultivation of *M. sextelata* provides a foundation for widespread cosumption (Li et al., 2023). With the achievement of large-sacle cultivation of morels, research on their bioactive components is also increasing. *M. sextelata* exhibits a morel-like upper cap structure that is dark brown to black in color, with deep pits and ridges that give it a honeycomb-like appearance. The cap of *M. sextelata* is supported by a dark brown to black hollow stem, which plays a crucial role in its commercial value. The darker the color of the cap, the more highly sought-after it is in the market. Pigment serves as the foundation for color development in edible fungi, such as nor-guanacastepene pigments in the Chilean mushroom *Cortinarius pyromyxa* (Lam et al., 2019), carotenoid in *Cordyceps militaris* (Dong et al., 2013), and melanin in *Pleurotus djamor* (Valdez-Solana et al., 2023). The color diversity in edible fungi is determined by the constituents of various pigments.

Melanins are ubiquitous in all biological kingdoms, yet their structure and function remain to be more comprehensively understood. Melanin is also a crucial natural pigment found in various edible fungi, including *P. citrinopileatus*, *P. cornucopiae* (Zhang et al., 2021), *Auricularia auricula* (Prados-Rosales et al., 2015). The melanin is characterized by a complex molecular structure resulting from the polymerization of phenolic and/or indole monomers. Furthermore, melanin primarily exists in three forms: eumelanin, allomelanin, and pheomelanin (Belozerskaya et al., 2017). In comparison to synthetic melanin, fungal natural melanin not only possesses environmentally friendly and biodegradable properties but also exhibits a range of exceptional functional characteristics and biological activities. Fungal melanin has been found to possess liver-protective, blood lipid-lowering, anti-cancerous, and immune-regulating effects that promote the maintenance of human health homeostasis (Liu et al., 2022). Therefore, it has extensive applications in various fields of daily life, such as dyeing, food production, and biotechnology. Furthermore, chemically modified fungal melanin exhibits stronger solubility and biological activity, thus presenting a wider range of potential applications. Despite the promising potential applications of fungal melanin, there are still challenges that need to be addressed. One primary obstacle is the low yield of fungal melanin production which limits its commercial viability. Another challenge lies in the incomplete understanding of fungal melanin biosynthesis. The regulation mechanism behind fungal melanin synthesis remains elusive and further research is necessary to elucidate the complete biosynthetic pathway (Eisenman and Casadevall, 2011). A comprehensive approach that addresses these challenges will be required for continued research and development of fungal melanin.

Light is a ubiquitous signal in the natural world, exhibiting variations in intensity and wavelength across different locations. Light signals play important roles in the biosynthesis of secondary metabolites and the ontogeny of fungi. For example, light can regulate *Aspergillus niger*’s biofilm formation by affecting melanin biosynthesis (Sun et al., 2021). The production of melanin in fungi is often associated with environmental stressors such as exposure to ultraviolet (UV) radiation or reactive oxygen species (ROS) (Eisenman et al., 2020). Melanin pigment can enhance fungal resistance to environmental damage. Its production has been shown to be important for maintaining fungal cell wall integrity, resisting oxidative stress, and promoting virulence (Gessler et al., 2014). Significant variation in cap color of *M. sextelata* was observed during the cultivation process across different regions. The fruiting body morphogenesis of morels is intricately and exquisitely connected with light influence. However, the effect of light environment on melanin synthesis in *M. sextelata* remains unclear.

The genetic basis and regulatory mechanism of the appearance and color of edible fungi have always been important scientific issues in the edible mushroom industry. Understanding these factors is crucial for improving the quality and market value of edible fungi. However, there is still limited research on how light affects the coloration of *M. sextelata*, as well as the specific types and structures of melanin present in its cap, which seriously restricts in-depth research on light environment regulation in *M. sextelata* and the utilization of its melanin pigments. In this study, our aim is to shed light on the effects of blue light on *M. sextelata*’s melanin pigments by providing a comprehensive structural characterization. The results will represent an important step towards unraveling the mysteries surrounding *M. sextelata*’s appearance and coloration through an exploration of its genetic basis and regulatory mechanism. Further comprehension of the biosynthesis and structural characteristics of melanin in *M. sextelata* can also facilitate its potential applications across various fields and enrich its theory on light environment control for high-quality cultivation.

## 2. Materials and methods

### 2.1. Strain, culture and sample preparation

The *M. sextelata* strain (CCMJ5600) was obtained from the Culture Collection Center of Mycology of Jilin Agriculture University. For the pure culture of *M. sextelata*, a punch (a diameter of 5 mm) from solid medium was inoculated onto the center of potato dextrose agar (Difco, Becton-Dickinson Co., Sparks, MD, United States) and incubated at 24°C for 5 days. Five 5 mm mycelial disks were inoculated into pre-culture spawn medium consisting of wheat (75.5%), rice husk (20%), humus (3%), superphosphate (1%) and lime (0.5%) in a polyethylene bag measuring 17 × 15 cm, and incubated at a temperature of 24°C for 8 days. Subsequently, the pre-culture spawn was inoculated into spawn medium containing wheat (60%), corn cob (18%), rice husk (10%), humus soil (10%), superphosphate (1%) and lime (1%). The nutrition bag formula used in the cultivation process was as follows: wheat (40%), corn cob (28%), rice husk (20%), humus (10%), superphosphate (1%). Each bag contained 1.5 kg of medium with 60% humidity. The cultivation process was completed in an artificial climate chamber, and the management methods refer to the previously published methods (Longley et al., 2019). During the primordial induction period, the light conditions included 6 h of supplemental lighting per day. After the fruiting body grows to 3 cm during the mushroom growing period, the lighting time was adjusted to 10 h per day. The light conditions utilized during the cultivation process were depicted in Supplementary Figure S1, and the proportion of blue light present under two distinct lighting regimes differed. There were two types of LED light strips, one with a low blue light ratio (LB) and the other with a high blue light ratio (HB). Both LED strips utilized the same light intensity of 20 μmol·m^−2^·s^−1^. Finally, the cap on the fruiting body was collected, dried in a vacuum freeze dryer (Christ ALPHA 1-2LD plus, Martin Christ Gefriertrocknungsanlagen GmbH, Osterode am Harz, Germany), and grounded into powder.

### 2.2. Visual colors of the cap under different blue light conditions

The cap color of *M. sextelata* grown under different blue light conditions was monitored using a portable colorimeter (Dongguan Zhongsheng Instrument Co., Ltd., Dongguan, Guangdong, China). Numeric description of the color was obtained using the L*, a* and b* CIELAB color space.

### 2.3. Isolation and purification of melanin

The melanin in the cap of *M. sextelata* was isolated and purified using a previously established milder protocol (d'Ischia et al., 2013).

### 2.4. Assessment of solubility and chemical reactivity of melanin pigment

The solubility and chemical reaction analysis of melanin pigment were conducted using the established method as previously described with minor modifications (Zhang et al., 2021). The solubility of the melanin pigment was assessed in various aqueous and organic solvents, including water, 0.5 M NaOH, 3 M HCl, ethanol, chloroform, methanol and ethyl acetate. Absorbance at 230 nm was measured to determine the degree of solubility.

### 2.5. Microscopic observations of melanin pigments

#### 2.5.1. Immunofluorescence

The melanin pigment present in the mycelia on the cap of *M. sextelata* was visualized using immunofluorescence staining, following a previously published protocol (Wong et al., 2018). Finally, the stained mycelia were observed using a laser scanning confocal microscopy (Zeiss LSM700, Jena, Germany).

#### 2.5.2. Microscopic analysis of melanin pigments with scanning electron microscopy (SEM) and transmission electron microscopy (TEM)

The melanin pigments and pilepellis of *M. sextelata* with different cap colors were separately observed using SEM and TEM techniques. The pileipellis of *M. sextelata* was carefully excised from the cap using a surgical blade and subsequently cut into 1 mm^2^ sections. The preparation of melanin samples for SEM followed a previously published protocol (Qiu et al., 2018). The sample was coated with a layer of gold by sputtering using an EMitech K550 sputter coater, and then observed under an HITACHI Regulus 8,100 scanning electron microscope at a voltage of 5 kV. The TEM samples were prepared following a previously established method (Yan et al., 2020). A Leica EM UC7 instrument was used to cut ultrathin (80 nm thick) sections. TEM observation was performed using a HITACHI HT7800/HT7700 transmission electron microscope operating at an acceleration voltage of 80 kV.

### 2.6. Physicochemical properties of melanin pigments

#### 2.6.1. UV–visible light absorption spectra

The absorption spectra of melanin pigments were detected using a previous established method with minor modifications (Rassabina et al., 2020). Briefly, the melanin pigment was solubilized in 0.1 M NaOH (50 mg/L). The absorption properties were measured in the UV–visible spectrum (200–1,100 nm) using quartz cuvettes with an optical path of 2 mm on a UV-1900 spectrophotometer (Macy Instruments Inc., Shanghai, China).

#### 2.6.2. Fourier transform infrared (FTIR) spectroscopy analysis

The FTIR spectra of melanin pigments were analyzed using a previously established method with minor modifications (Ghadge et al., 2023). Pure KBr powder and melanin pigment were homogenized in an agate mortar, followed by mixing 10 mg of melanin pigment with 200 mg of KBr. The resulting mixture was then pressed into transparent sheets (7 mm) using a Mini-Pellet Press (Yingshite Instrument Technology Co., Ltd., Suzhou, China). Melanin-KBr pellets were analyzed using a Nicolet^™^ iS 50 FTIR (Thermo Fisher Scientific, Madison, United States) in the wave range of 4,000–400 cm^−1^ with a resolution of 4 cm^−1^. Each spectrum was an average of 32 scans, and KBr spectra were recorded as background.

#### 2.6.3. Solid-state nuclear magnetic resonance (NMR) spectroscopy

The solid-state ^13^C NMR spectra were obtained using a Bruker 400 MHz AVANCE III spectrometer euipped with a 4 mm H-X-Y MAS probe (BrukerBioSpin, Rheinstetten, Germany), following the established methodology (Zhang et al., 2021).

#### 2.6.4. Elemental composition analysis

The elemental compositions of melanin pigments was analyzed using single quadrupole inductively coupled plasma mass spectrometry (SQ-ICP-MS) from Thermo Fisher Scientific (Potočnik et al., 2021). The proportions of carbon (C), hydrogen (H), oxygen (O), nitrogen (N), and sulfur (S) present in the melanin pigment were determined.

#### 2.6.5. High performance liquid chromatography (HPLC) analysis

The melanin pigments of *M. sextelata* were analyzed using the HPLC method and compared with standard eumelanin (M8631, Sigma-Aldrich, St. Louis, MO, United States) (Ma et al., 2023). The pigments and the standard eumelanin were dissolved in a 0.5 M NaOH solution at a final concentration of 50 mg/L. The chromatographic analysis was conducted using an Agilent 1,290 HPLC system (Agilent Technologies, Inc., Santa Clara, CA, United States) with a Waters C18 column (300 mm × 7.8 mm, 5 μm, Milford, MA, United States). The mobile phases consisted of 1% acetic acid (pH 2.8) and methanol in a volume ratio of 97:3, with a flow rate of 0.2 mL/min and an injection volume of 20 μL. Detection was performed at a wavelength of 210 nm, while the column temperature was maintained at 25°C.

### 2.7. Statistical analysis

The analyzes were conducted in triplicate, and the results were reported as mean ± standard deviation (SD). The one-way analysis of variance (ANOVA) with Duncan’s multiple range tests was employed to assess and compare the significant differences among the various samples. Statistical analysis was performed using SPSS software (version 19.5, IBM SPSS Statistics, Armonk, NY, United States).

## 3. Results and discussion

### 3.1. Detection and quantification of melanin pigments production by *Morchella sextelata*

**Figure 1:**
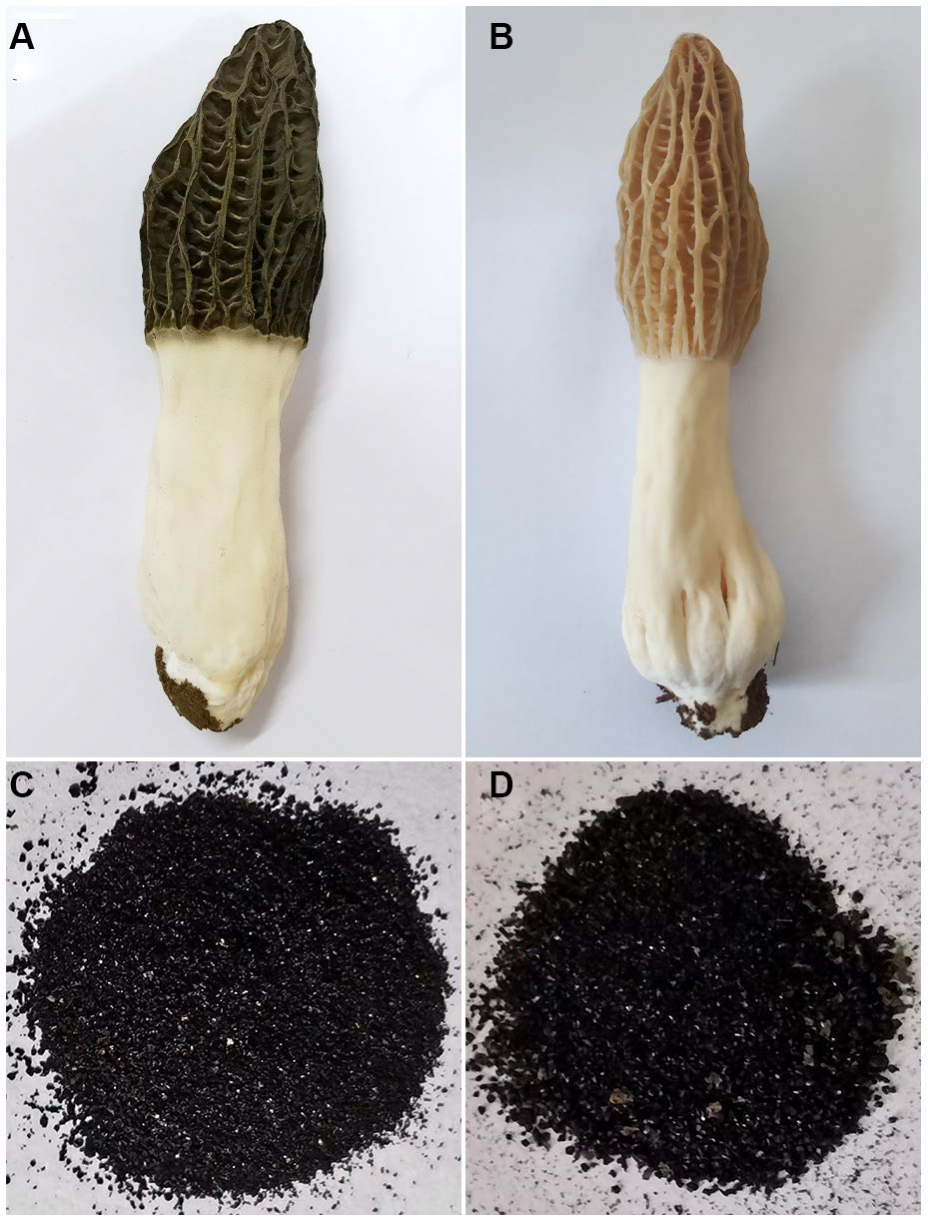
Appearance characteristics of the fruiting body and melanin pigments. **(A)** The fruiting body of *M. sextelata* cultivated under high blue light ratio light conditions. **(B)** The fruiting body of *M. sextelata* cultivated under low blue light ratio light conditions. **(C)** Melanin pigment extracted from the black cap of *M. sextelata*. **(D)** Melanin pigment extracted from the brown cap of *M. sextelata*.

**Figure 2:**
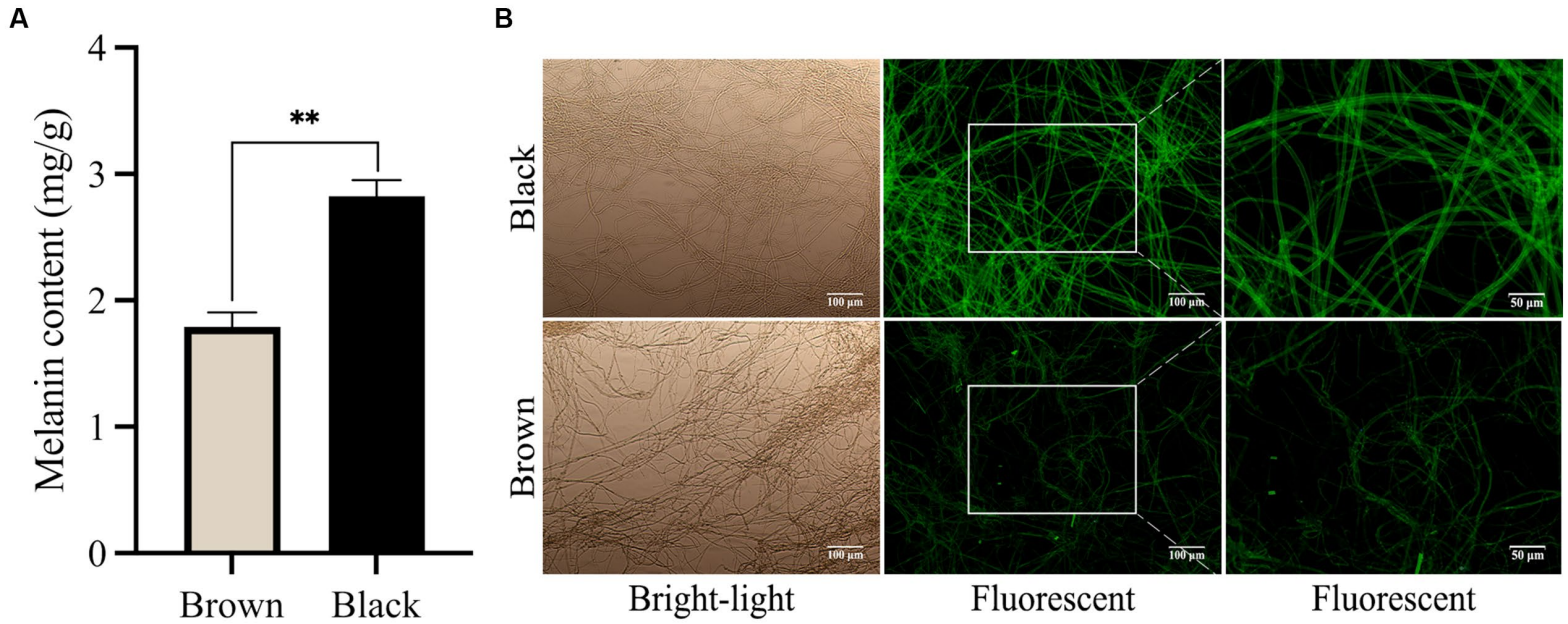
Blue light promotes the production of melanin. **(A)** The extracted melanin pigment content from caps of varying hues. **(B)** Microscopic images of mycelium in different colored cap of *M. sextelata*. The values represent the means and standard deviations of three independent experiments, with statistical significance at *p* < 0.01 using Student’s *t*-test.

There is a lack of analytical reports on the optimal or favorable usage of LEDs for cultivating *M. sextelata* in facilities, particularly regarding the optimization of light conditions such as wavelength, intensity, and irradiation time. In the present study, light wavelengths showed considerable influence on the cap color of *M. sextelata*. The fruiting body cap color exhibited significant differences when cultivated under different spectral irradiation. As shown in Figures 1A,B, the cap of *M. sextelata* cultivated under low blue light ratio (LB) conditions exhibited a brown hue, while the cap color was notably blackened in high blue light environments ratio (HB). Under the LB treatment, the L* value of the fruiting bodies’ cap was found to be significantly higher than that observed under HB treatment, indicating a lower degree of blackening. Conversely, fruiting bodies grown under HB treatment exhibited significantly lower L* values (Supplementary Table S1). Moreover, the b* value indicating the level of yellow saturation was significantly higher in brown-capped specimens than in black-capped ones. This phenomenon has also been observed in *Inonotus obliquus*, where exposure to blue light can stimulate melanin synthesis (Poyedinok et al., 2015). Light signaling pathways are intricately interconnected with other signaling cascades, developmental pathways, and metabolic networks (Yu and Fischer, 2018). This could be attributed to the activation of melanin synthesis pathways with high expression levels in cells by blue light signals. Similar to the color of the cap, melanin extracted from the brown cap appeared as a brownish black hue, while that extracted from the black cap was characterized by a black pigment (Figures 1C,D). The melanin content extracted from the darker cap was found to be as high as 2.82 mg/g (the weight of pure pigment per 1 g dry cap powder), which was significantly (*p* < 0.01) higher than that of the lighter brown cap (Figure 2A). The high melanin content extracted from the darker cap of *M. sextelata* not only demonstrates its potential as a valuable natural pigment but also highlights its superiority over other fungal species such as *A. auricula* in terms of melanin production (Islam et al., 2022). Fungal melanin is a promising natural pigment with a wide range of potential applications in various fields such as medicine, agriculture, material science, and cosmetics. The unique properties of fungal melanin make it an attractive candidate for numerous applications. The findings of the present study indicate that *M. sextelata* has significant potential as a functional food source for melanin acquisition. Furthermore, adjusting and increasing the proportion of blue light in its light environment can promote melanin accumulation in the cap of *M. sextelata*, enhancing both its commercial quality and biological activity.

### 3.2. Microscopic analysis of melanin pigments

#### 3.2.1. Immunofluorescence staining analysis

As shown in Figure 2B, the visualization of the melanin pigment in the mycelia through immunofluorescence staining provided valuable insights into its specificity and sensitivity. This technique allowed us to observe a high level of fluorescence signals within the mycelium of *M. sextelata*, particularly in the black cap region. Interestingly, our findings align with a previous study in *A. auricula* (Sun et al., 2021), which established a positive correlation between the intensity of fluorescence and the quantity of melanin present. This correlation further supports our observation that an increase in blue light proportion within the light environment stimulates melanin accumulation on the surface of *M. sextelata*’s mycelium.

#### 3.2.2. Ultrastructural characteristics of melanin pigments

**Figure 3:**
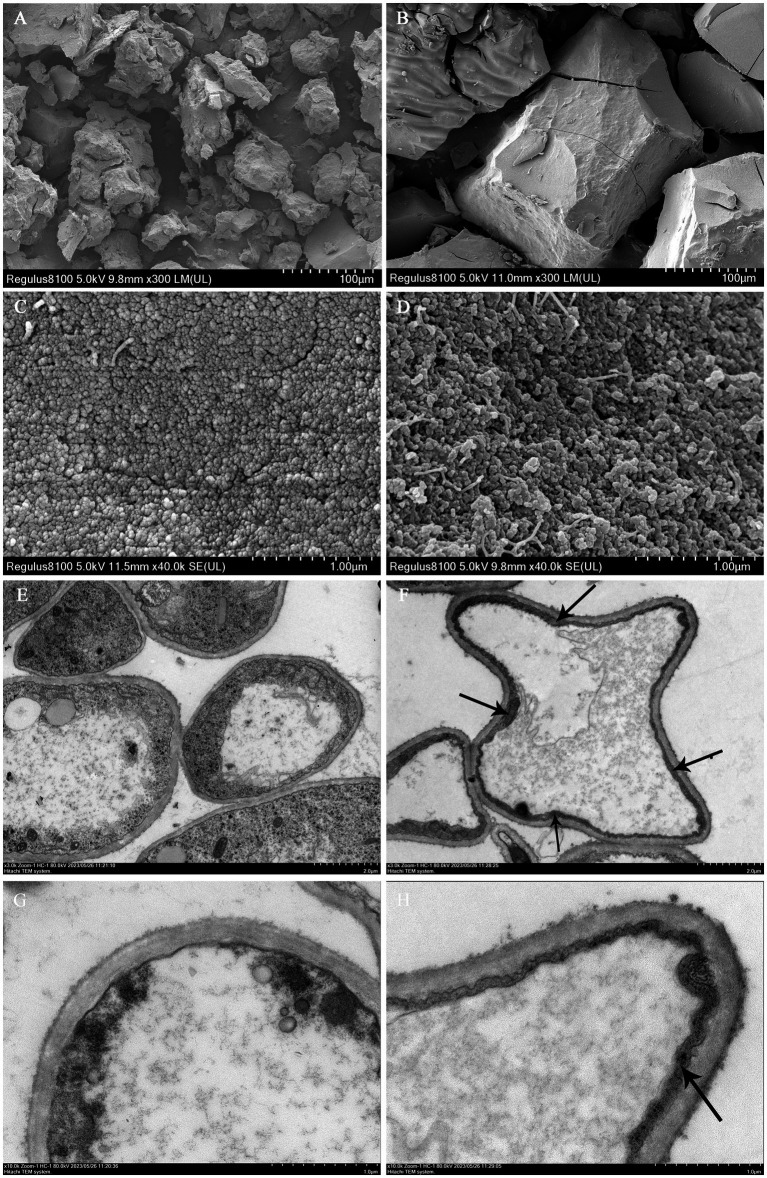
Ultrastructure micrographs of mycelium and melanin pigments. SEM images of melanin pigment extracted from the brown cap **(A,C)** and the black cap **(B,D)**. TEM observations of the mycelia in the brown cap **(E,G)** and black cap **(F,H)**.

The precise localization of melanin in the mycelia and ultrastructural characteristics of melanin were analyzed using SEM and TEM techniques. Melanin pigments in fungi can be detected within the cell wall and observed as a dark substance under microscopic examination (Kogej et al., 2008). The SEM micrographs revealed that both types of melanin pigments consist of amorphous materials composed of irregularly aggregated numerous small spherical granules ranging in diameter from 100 to 300 nm (Figures 3A–D). The results were consistent with the previously observed presence of melanin pigment in *Mycosphaerella fijiensis* (Beltran-García et al., 2014). These granules were located beneath the polysaccharide capsule and adjacent to the plasma membrane. Prior studies on the microstructure of melanins associated with the cell wall had revealed that they were composed of multiple layers of spherical granules ranging in diameter from 50 to 80 nm (Eisenman et al., 2009). However, the morphology and dimensions of melanin granules often exhibit variations depending on their origin (Pralea et al., 2019). TEM observation revealed that compared to the brown cap, the mycelia in black cap harbored a thick and rough inner layer of the cell wall (Figures 3F,H, arrow), indicating a substantial accumulation of melanin within the innermost region of the cell wall compared to those in brown cap (Figures 3E,G). Moreover, it was an internal electron-dense material layer. Previous research has demonstrated that the distribution of melanin within fungal cell walls exhibits species-specific variation. For instance, melanin synthesized through the _L_-3,4-dihydroxyphenylalanine (_L_-DOPA) pathway is located in the innermost layer of the cell wall adjacent to the plasma membrane in *Cryptococcus neoformans* (Nosanchuk and Casadevall, 2003). The comprehensive research findings suggest that blue light can stimulate melanin synthesis by activating the _L_-DOPA pathway and significantly accumulate in the inner layer of cell walls, ultimately resulting in a significant darkening of cap color.

### 3.3. Solubility and chemical reaction analysis of melanin pigments

The solubility of melanin pigments from caps of various colors in *M. sextelata* was determined. As shown in Supplementary Table S2, the extracted melanin was essentially insoluble in water, HCl, and various organic solvents tested, exhibiting negligible absorbance at 230 nm. However, it was soluble in NaOH solution with an absorbance value exceeding 1.5. The findings indicate that the melanin pigment is insolubility in water, HCl and measured organic solvents but soluble in NaOH solution. Furthermore, the pigment underwent bleaching upon reaction with the oxidizing agent H_2_O_2_, while displaying no reactivity towards the reducing agent Na_2_SO_3_. Brown precipitates were formed upon reaction with FeCl_3_ (Supplementary Table S3). This further supports our understanding of the properties and behavior of this particular type of melanin pigment. Based on this part of the results, it was found that the melanin in *M. sextelata* is similar to that in *P. cornucopiae* (Zhang et al., 2021) and *A. auricula* (Sun et al., 2016), possibly due to their similarity in structure and composition.

### 3.4. Elemental composition of melanin pigments

Previous studies have provided evidence that melanin, the pigment responsible for coloration in organisms, is a complex polymer composed of different types such as eumelanin (which contains 8.49% N and 0.09% S), pheomelanin (which contains 9.36% N and 9.78% S), and typical allomelanin (which contains no N) (Sun et al., 2016). These melanins differ in their elemental composition and play crucial roles in determining the color patterns observed in various species. In this study, elemental composition analysis of melanin pigments revealed the presence of elements C, N, H, O, and S in both samples (Table 1). The melanin pigment extracted from the brown cap contained 49.99% C, 6.85% H, 7.27% N, 0.71% S and 35.18% O, whereas that from the black cap contained 53.55% C, 5.84% H, 9.28% N, 1.23% S and 30.10% O. The S element content of melanin in the brown cap falls within the range between eumelanin and pheomelanin, with a value of 0.78%. Therefore, we can speculate that the melanin present in the brown cap comprises both eumelanin and pheomelanin. However, the N element content in the brown cap melanin was only 7.27%, significantly lower than that of eumelanin and pheomelanin. This observation leads us to speculate that allomelanin may still be present within the brown cap’s pigment composition despite its lower overall contribution to nitrogen content. Furthermore, there was a significant increase in the S element content within the black cap, indicating an elevation in pheomelanin levels. There are two main pathways for synthesizing melanin in fungi: the 1,8-dihydroxynaphthalene (DHN) pathway and the _L_-DOPA pathway. Allomelanin is synthesized *via* the DHN pathway, while eumelanin and pheomelanin are synthesized through the _L_-DOPA pathway (Eisenman and Casadevall, 2011). Our research findings validate the presence of both DHN and _L_-DOPA pathways for synthesizing allomelanin, eumelanin, and pheomelanin in *M. sextelata*. Furthermore, an increase in blue light exposure within the environment can stimulate pheomelanin production via the _L_-DOPA pathway, resulting in a shift in cap coloration for *M. sextelata*.

**Table 1:** Elemental composition of melanin pigments extracted from caps of *M. sextelata* with different colors.

Melanin pigments	Content (%)				
	C	H	N	S	O^a^
Brown cap	49.99	6.85	7.27	0.71	35.18
Black cap	53.55	5.84	9.28	1.23	30.10

a The content of O element was calculated from the equation: O% = 100%-C%-H%-N%-S%.

### 3.5. Spectral analysis of melanin pigments

#### 3.5.1. UV–visible light absorption spectrum

**Figure 4:**
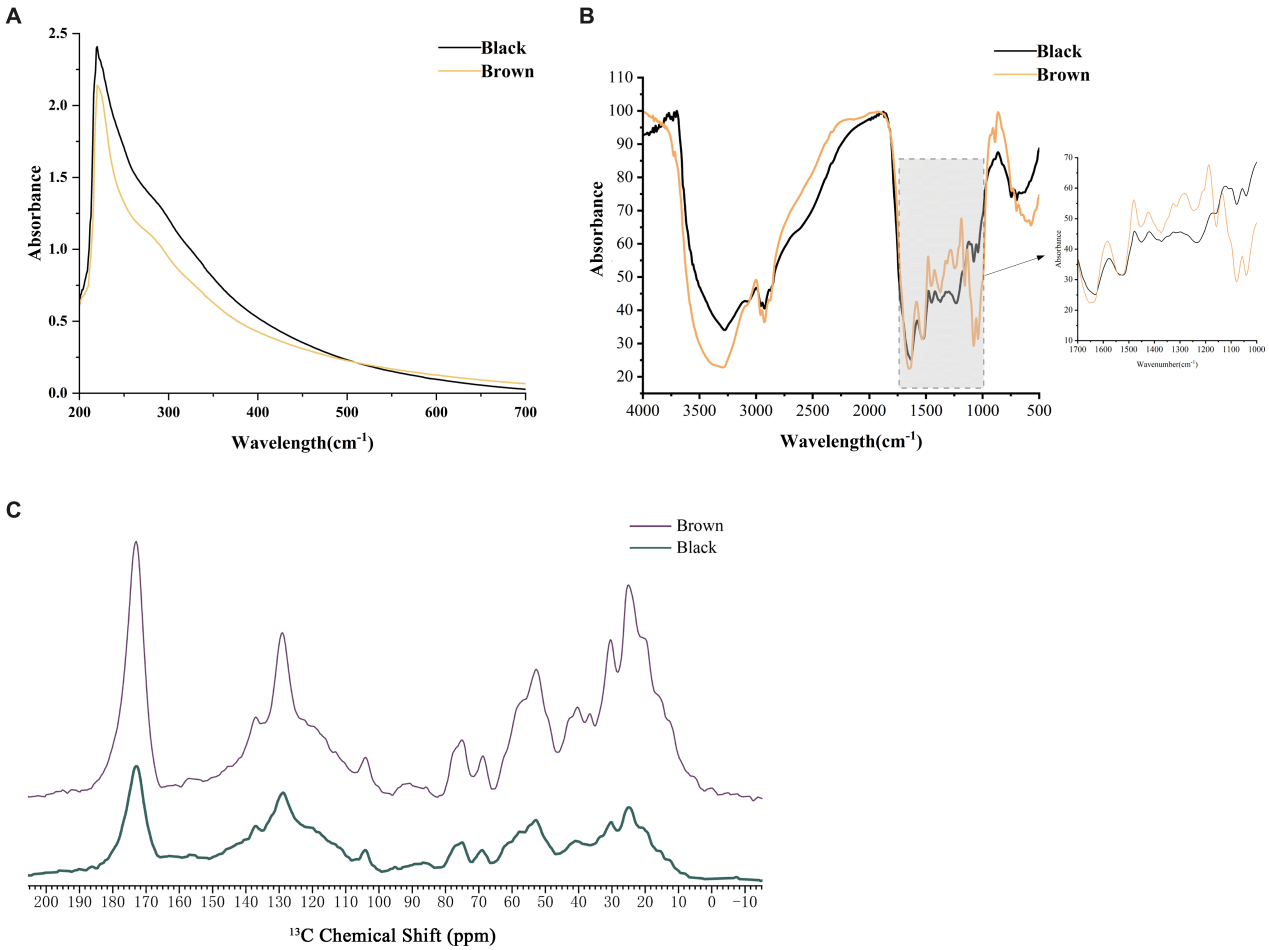
Spectral analysis of melanin pigments extracted from caps of varying colors. **(A)** UV–visible spectra of melanin pigment. **(B)** FTIR spectra of melanin pigment. **(C)** NMR spectra of melanin pigment. Black represented the melanin extracted from the black cap. Brown represented the melanin extracted from the brown cap.

Melanin pigments are known for their remarkable ability to absorb a wide range of light wavelengths. In the study, it was observed that both types of melanin exhibited strong absorbance in the UV region, with a gradual decrease in optical density as the wavelength increased (Figure 4A). The maximum absorption peak of the two melanin pigments in *M. sextelata* was observed at 220 nm in the UV–visible absorption spectrum. The results presented herein are consistent with the commonly observed UV–visible absorption characteristics of typical melanin, although they show slight deviations at the maximum absorption peak (Ma et al., 2023). These variations in maximum absorbance could be attributed to differences in the source of melanin, which may result in minor alterations to its natural structure. It is fascinating how even small changes can influence such fundamental properties. Furthermore, no additional absorption peaks were observed at 260 and 280 nm, indicating the absence of nucleic acids, lipids, and other proteins in the melanin pigments isolated from *M. sextelata*. Understanding the unique characteristics and properties of melanin pigments is crucial not only for scientific research but also for various practical applications.

#### 3.5.2. FTIR analysis of melanin pigments

FTIR is a widely utilized spectroscopic technique for the investigation and characterization of melanin pigment (Khouqeer et al., 2022). Both melanin pigments showed similar characteristic peak absorption spectra in the FTIR analysis (Figure 4B; Supplementary Table S4). The absorption band observed at 3282 cm^−1^ and 3,008 cm^−1^ exhibited the characteristic of stretching vibration modes for O-H or N-H bonds. It may result from the stretching vibrations of O-H and N-H in carboxylic acid, phenolic compounds, as well as aromatic amino groups present in indole and pyrrole systems (Zhang et al., 2021). Peaks at 2929 cm^−1^ and 2,872 cm^−1^ indicated the stretching vibration characteristic of aliphatic C-H group (-CH_2_ and -CH_3_). The melanin pigments also exhibited absorption band at 1654 cm^−1^ and 1,540 cm^−1^, indicating the presence of bending vibrations modes of the aromatic ring C=C and C=N bond. The peak at 1596 cm^−1^ represented an overlapping O-H (def) of the C=C ring stretching. A peak at 1523 cm^−1^, attributed to the bending vibration of N-H and the stretching vibration of C-N (secondary amine), can be observed in *A. niger* (Pralea et al., 2019). The peaks observed at 1457 cm^−1^ and 1,421 cm^−1^ were indicative of the bending vibrations modes associated with aliphatic C-H groups (-CH, -CH_2_ and -CH_3_), while the peak detected at 1378 cm^−1^ corresponded to the presence of a C=O or COO- group. The observed peaks at 1252 cm^−1^, 1,159 cm^−1^, and 1,078 cm^−1^ indicated the presence of alcoholic C-O or C-H in-plane vibrations within the aliphatic structure. *M. sextelata* melanin pigments also showed characteristic bands for aromatic rings and sulphur at 730 cm^−1^ and 678 cm^−1^. Comparative results between *M. sextelata* melanin pigments and those found in other organisms such as *A. heimuer* (Ma et al., 2023), and oyster mushroom (Zhang et al., 2021), indicate a similarity in their characteristic peak absorption spectra obtained through FTIR analysis. This similarity further supports the notion that there might be commonalities in terms of melanin composition among different species.

#### 3.5.3. NMR spectroscopy of melanin pigments

Due to the heterogeneous and amorphous nature of melanin pigments, as well as their insolubility in aqueous buffers, specialized techniques such as solid-state NMR are necessary for elucidating the constituent chemical moieties present within the intact pigment (Khouqeer et al., 2022). As shown in Figure 4C, the melanin pigments extracted from *M. sextelata* exhibit ^13^C NMR spectra featuring signals corresponding to aromatic and/or olefinic carbon resonances (110–160 ppm), long-chain aliphatic methylene groups [(CH_2_)_n_, 20–40 ppm], key oxygenated aliphatic carbon moieties (CH_2_O, 62 ppm; CHO, 50–105 ppm), and carboxyl or amide groups (COO or CONH, 173 ppm). The peaks observed within the 40–60 ppm range can be attributed to the presence of carbon or CH-N/CH-S moieties (Palani and Gandhi Shree, 2021). The presence of resonances at 30 ppm, 24 ppm, and 15 ppm indicated a characteristic pattern of alkyl chains consisting of (CH_2_)_n_CH_2_CH_3_. The resonance spectrum observed at 128 ppm may be attributed to the overlapping resonances of aromatic indole-based constituents. Comparative analysis with oyster mushroom melanin pigments using solid-state NMR provids evidence for similarities in carbon-containing structures between *M. sextelata* and oyster mushroom species (Zhang et al., 2021).

### 3.6. Composition analysis of melanin pigments in caps of different colors

**Figure 5:**
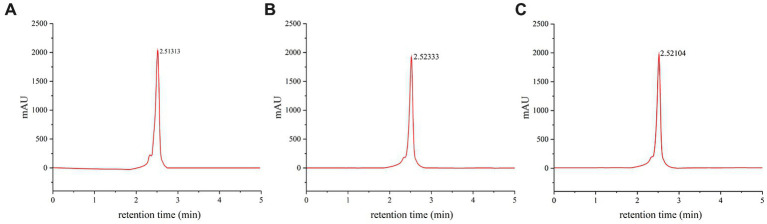
Component analysis of melanin pigments. HPLC chromatogram of standard eumelanin **(A)**, melanin pigment extracted from the brown cap of *M. sextelata* **(B)**, and melanin pigment extracted from the black cap of *M. sextelata* **(C)**.

The variation in combination and proportion of different types of melanin often results in a diverse range of hues in edible fungi (Lin and Xu, 2022). This diversity in coloration has intrigued scientists for many years, leading to the development of various methods to analyze the composition and proportion of melanin pigments. In order to improve the analysis process, a standardized eumelanin and HPLC method was employed (Figure 5A). HPLC is a powerful technique that allows for the separation and identification of different compounds within a sample. In this case, it was used to separate and quantify melanin pigments present in the caps of *M. sextelata*. The chromatographic profile of melanin pigments appeared as a single, symmetrical elution peak. The melanin pigments extracted from caps of different colors exhibited a single symmetrical elution peak in the chromatogram profile, with a retention time of 2.52 min, which was identical to that of standard eumelanin (Figures 5B,C). According to HPLC analysis, the melanin pigments extracted from the brown cap exhibited a eumelanin content of 46.4%, whereas those extracted from the black cap demonstrated a higher concentration of 49.1%. The significantly higher total melanin content extracted from the black cap suggests a significantly greater amount of eumelanin in the black cap compared to that in the brown cap. Additionally, combined with the preceding elemental composition analysis, it has been confirmed that blue light possesses the capability to stimulate pheomelanin synthesis. Furthermore, based on HPLC results, it has also been established that blue light can promote eumelanin synthesis. Based on the previous data, it can be confirmed that blue light has the capacity to stimulate eumelanin and pheomelanin synthesis via the _L_-DOPA pathway, leading to a darker cap color of *M. sextelata*. Fungi are capable of sensing alterations in light wavelength and intensity via photoreceptors, which subsequently transmit light signals to cells for the regulation of gene expression. This enables fungi to adapt to environmental changes or pressures (Yu and Fischer, 2018). The mechanism of gene activation in response to blue-light exposure has been extensively investigated in fungi (Sancar et al., 2015). However, there have been no reports on the promotion of fungal melanin synthesis by blue light. The present study has made an innovative discovery, revealing the significant impact of blue light on melanin synthesis in *M. sextelata*.

## 4. Conclusion

In this study, we present the first evidence of the impact of blue light on the cap color of *M. sextelata*. The results showed that blue light played a crucial role in triggering the synthesis of melanin pigments in *M. sextelata*. We identified and characterized the melanin present in *M. sextelata*, and found that both brown and black caps contain three distinct types of melanin: eumelanin, pheomelanin, and allomelanin. Furthermore, the structural characteristics of the melanin pigments were examined using various spectroscopic and microscopic techniques. The results revealed that the melanin compounds exhibited a complex arrangement of polymeric structures with evident variations in their chemical compositions. This is the first instance where a correlation between blue-light signaling and melanin has been established in *M. sextelata*. To our knowledge, the promotion of melanin synthesis in fungi through blue-light signaling is also a novel discovery. The findings suggest that the biosynthesis of melanin in *M. sextelata* is primarily regulated by photoinduction mechanisms associated with blue light. The results shed light on the potential applications of blue-light signaling for cultivating high-quality *M. sextelata* fruiting bodies. The results will facilitate researchers in delving deeper into understanding the molecular mechanisms underlying melanin synthesis and its regulation by blue-light signaling, thereby establishing a solid theoretical foundation for further research into the photophysiology of *M. sextelata* as well as opening up new avenues to explore similar light regulation mechanisms underlying color formation in other edible mushrooms.

## Data availability statement

The original contributions presented in the study are included in the article/Supplementary material, further inquiries can be directed to the corresponding author.

## Author contributions

ZQ: Conceptualization, Data curation, Formal analysis, Funding acquisition, Methodology, Software, Writing – original draft. SW: Data curation, Formal analysis, Methodology, Software, Writing – original draft. JZ: Writing – original draft, Data curation, Formal analysis, Investigation, Methodology, Software. LC: Writing – original draft, Data curation, Formal analysis, Investigation, Software. XW: Data curation, Formal analysis, Investigation, Software, Writing – original draft. NC: Data curation, Investigation, Software, Writing – original draft. HL: Data curation, Investigation, Software, Writing – original draft. SR: Data curation, Investigation, Software, Writing – original draft. TL: Investigation, Validation, Visualization, Writing – original draft. LS: Conceptualization, Formal analysis, Funding acquisition, Investigation, Methodology, Project administration, Resources, Supervision, Visualization, Writing – review & editing.

## Funding

The author(s) declare financial support was received for the research, authorship, and/or publication of this article. This work was supported by Project funded by China Postdoctoral Science Foundation (grant number: 2023MD734195), Science and Technology Special Mission Project of Chaoyang County (grant number: Z20220224).

## Conflict of interest

The authors declare that the research was conducted in the absence of any commercial or financial relationships that could be construed as a potential conflict of interest.

## Publisher’s note

All claims expressed in this article are solely those of the authors and do not necessarily represent those of their affiliated organizations, or those of the publisher, the editors and the reviewers. Any product that may be evaluated in this article, or claim that may be made by its manufacturer, is not guaranteed or endorsed by the publisher.
