# Supplementary material for: Synthesis and structural characteristics analysis of melanin pigments induced by blue light in Morchella sextelata
Source: Front Microbiol. 2023 Sep 29;14:1276457. doi: 10.3389/fmicb.2023.1276457 (PMC10573313; doi:10.3389/fmicb.2023.1276457)
Supplement: Supplementary file 1 [file Data_Sheet_1.docx]

Supplementary Material

# Supplementary Figures and Tables

## Supplementary Figures


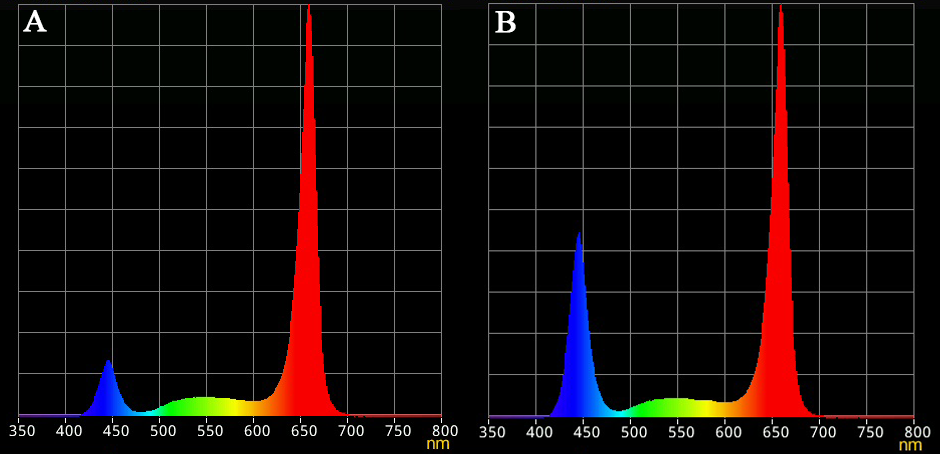


**Supplementary Figure 1.** Spectral composition of LED light strips for the cultivation of *M. sextelata*. **(A)** The LED light strips featuring a low blue light ratio. **(B)** The LED light strips featuring a high blue light ratio.

## Supplementary Tables

Table S1. Color analysis of the cap in *M. sextelata*

| Treatments | Color reading | | |
| --- | --- | --- | --- |
|  | *L** | *a** | *b** |
| LB | 41.46^a^ | 3.38^a^ | 15.75^a^ |
| HB | 17.23^b^ | 2.38^b^ | 5.26^b^ |

Note: The color is described numerically using the *L***a***b** CIELAB color space, where *L** (lightness or darkness) values range from black (0) to white (100), *a** represents the red-green direction (*a** > 0 for red and *a** < 0 for green), and *b** represents the yellow-blue direction (*b** > 0 for yellow and *b** < 0 for blue). Values with different superscript uppercase letters within the same column are significantly different at *p* ≤ 0.05.

Table S2. Measurement of the absorbance spectra of melanin pigments extracted from caps of varying colors in water, NaOH, HCl and common organic solvents

| Treatments | Absorbance | |
| --- | --- | --- |
|  | Brown | Black |
| Water | 0.009 | 0.012 |
| NaOH | 1.743 | 1.824 |
| HCl | 0.012 | 0.028 |
| ethanol | 0.022 | 0.034 |
| methanol | 0.033 | 0.034 |
| ethyl acetate | 0.022 | 0.020 |
| chloroform | 0.013 | 0.018 |

Table S3. Solubility and chemical reactivity assessments of melanin pigments derived from caps of varying colors

| Treatments | Results | |
| --- | --- | --- |
|  | Brown | Black |
| Water | Insoluble | Insoluble |
| Organic solvents | Insoluble | Insoluble |
| NaOH | Soluble | Soluble |
| HCl | Precipitate | Precipitate |
| H_2_O_2_ | Decolorized | Decolorized |
| Na_2_SO_3_ | - | - |
| FeCl_3_ | Brown precipitate | Brown precipitate |

Table S4. Spectral positions, center positions and corresponding assignments of FTIR peaks

| Peaks | Center [cm−1] | Assignments |
| --- | --- | --- |
| 1 | 3282, 3008 | Stretching vibration modes for O-H or N-H bonds |
| 2 | 2929, 2872 | Stretching vibration of aliphatic C-H group (C-H2 and C-H3) |
| 4 | 1654, 1540 | Bending vibrations modes of aromatic ring C=C and C=N bond of aromatic |
| 5 | 1586 | Overlapping O-H (def)of C=C ring stretching |
| 6 | 1523 | Bending vibration of N‐H and stretching vibration of C‐N (secondary amine) |
| 7 | 1457, 1421 | Bending vibration of C-H groups (CH, CH2 and CH3） |
| 8 | 1378 | C=O ＆COO- group |
| 9 | 1252, 1159, 1078 | Alcoholic C-O; C-H in-plance of aliphatic structure |
| 10 | 730, 678 | Weak absorption indicationg C-S stretching |
