# Supplementary figures and images for: Synthesis and structural characteristics analysis of melanin pigments induced by blue light in Morchella sextelata
Source: Front Microbiol. 2023 Sep 29;14:1276457. doi: 10.3389/fmicb.2023.1276457 (PMC10573313; doi:10.3389/fmicb.2023.1276457)

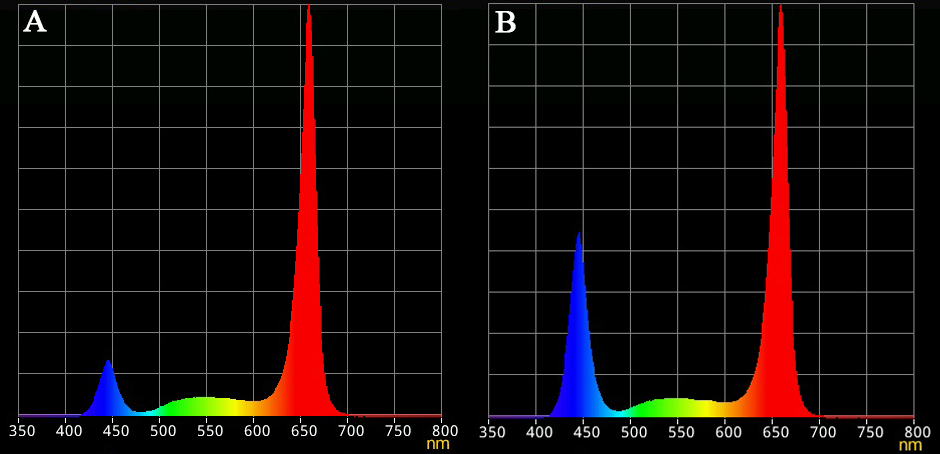

Supplement: Supplementary file 2 [file Image_1.TIF]
